# Tooth Loss and Cardiovascular Risk Burden in Myocardial Infarction Patients

**DOI:** 10.3390/jcm14228227

**Published:** 2025-11-20

**Authors:** Corina Cinezan, Camelia Bianca Rus, Alexandra Cinezan, Luminita Ligia Vaida

**Affiliations:** 1 Department of Medical Disciplines, Faculty of Medicine and Pharmacy, University of Oradea, 410073 Oradea, Romania; rus.cameliabianca@student.uoradea.ro; 2 Clinical County Emergency Hospital Bihor, 410169 Oradea, Romania; 3 Doctoral School of Biological and Biomedical Sciences, University of Oradea, 410087 Oradea, Romania; 4 Faculty of Dental Medicine, University of Medicine and Pharmacy, 400012 Cluj-Napoca, Romania; cinezan.alexandra@elearn.umfcluj.ro; 5 Department of Dental Medicine, Faculty of Medicine and Pharmacy, University of Oradea, 410068 Oradea, Romania; ligia_vaida@yahoo.com

**Keywords:** tooth loss, myocardial infarction, oral health, smoking, diabetes mellitus, atherosclerosis, cardiovascular risk, interdisciplinary care, secondary prevention

## Abstract

**Background:** Oral and cardiovascular health share inflammatory and behavioral pathways. Chronic oral inflammation leading to tooth loss may reflect systemic vascular injury. The relationship between tooth loss and cardiovascular risk among patients with myocardial infarction (MI) remains underexplored. **Objective:** We hypothesized that in post-MI patients, the extent of tooth loss reflects cumulative systemic inflammatory exposure and correlates with major cardiovascular risk factors and coronary artery disease severity. **Methods:** In this cross-sectional study, 200 consecutive MI patients (mean age 64 years, 65% male) underwent oral examination and cardiovascular risk assessment. Logistic and robust linear regression models were applied to identify independent predictors of tooth loss. **Results:** The median number of missing teeth was 20 (IQR 12–28). Age (OR 1.13/year, *p* < 0.001; β = 0.53, *p* < 0.001) and smoking (OR 3.28, *p* = 0.007; β = 3.13, *p* = 0.027) were significant independent predictors. Diabetes showed a borderline association (OR 2.20, *p* = 0.054), and a trend was observed with the number of affected coronary arteries (β = 1.05, *p* = 0.063). **Conclusions:** Tooth loss in MI patients is closely associated with age and smoking and may indicate cumulative inflammatory burden. As a simple, non-invasive marker, tooth loss could aid cardiovascular risk stratification and encourage interdisciplinary prevention integrating cardiology and dental care.

## 1. Introduction

Cardiovascular disease (CVD) remains the leading cause of morbidity and mortality worldwide, with myocardial infarction representing one of its most severe clinical manifestations [1]. Classical risk factors such as smoking, diabetes mellitus, hypertension, dyslipidemia, obesity, and advanced age are well established in the pathogenesis of atherosclerosis, the underlying process leading to coronary artery disease. However, in recent decades, there has been growing interest in the potential contribution of oral health, and particularly tooth loss, as a marker and possible mediator of cardiovascular risk [2,3].

Tooth loss is the final stage of chronic oral conditions, most commonly periodontal disease and dental caries. Periodontal disease is a chronic inflammatory condition caused by bacterial plaque accumulation and host immune response, which can result in progressive destruction of the supporting tissues of the teeth [4]. Severe periodontitis has been identified as a global public health problem, affecting more than 10% of the adult population, and is one of the leading causes of tooth loss. Beyond local consequences, periodontal disease has systemic effects, as bacteria and inflammatory mediators may enter the circulation, contributing to endothelial dysfunction, systemic inflammation, and atherosclerosis [5].

Epidemiological studies have shown associations between poor oral health and cardiovascular disease. Individuals with significant tooth loss or periodontitis have been reported to have a higher prevalence of hypertension, diabetes mellitus, and dyslipidemia, as well as increased risk of coronary heart disease and stroke [1,6,7]. Moreover, tooth loss is often considered a surrogate marker of lifetime oral disease burden and may reflect long-standing exposure to systemic inflammatory processes [4]. Nevertheless, the exact nature of the association between oral health and cardiovascular outcomes remains debated, with questions about causality, shared risk factors, and confounding by socioeconomic status or health behaviors [2].

In patients who have already suffered an MI, secondary prevention and risk stratification are crucial. Identifying novel and easily measurable indicators of increased atherosclerotic burden, such as the number of missing teeth, could provide additional insight into residual cardiovascular risk. Tooth loss is a simple and objective clinical parameter that may reflect lifelong exposure to systemic inflammation and behavioral risk factors. Yet, its relationship with classical cardiovascular risk factors and angiographically assessed disease severity among MI patients remains insufficiently explored. Examining the relationship between tooth loss and classical risk factors in post-MI patients can improve our understanding of common pathogenic mechanisms linking oral and cardiovascular health [8,9,10].

Recent evidence supports that periodontal inflammation contributes to systemic endothelial dysfunction and plaque instability through inflammatory mediators such as C-reactive protein (CRP), interleukin-6 (IL-6), and tumor necrosis factor-α (TNF-α) [11,12]. Moreover, socioeconomic and behavioral determinants—including education, oral hygiene habits, and access to dental care—modulate both oral disease severity and cardiovascular outcomes. Understanding the relationship between tooth loss and cardiovascular risk in post-MI patients may therefore provide a low-cost marker of cumulative inflammatory burden and help identify individuals requiring closer multidisciplinary follow-up.

Despite growing evidence linking oral inflammation with systemic vascular disease, few studies have examined this relationship specifically in post-MI populations, particularly in Eastern European cohorts where both oral disease and cardiovascular burden remain high [13].

Therefore, we hypothesized that in patients with myocardial infarction in our country, the extent of tooth loss reflects cumulative systemic inflammatory exposure and is associated with a higher prevalence of major cardiovascular risk factors and greater coronary artery disease burden.

To test this hypothesis, we analyzed the relationship between the number of missing teeth and established cardiovascular risk markers—including smoking, diabetes, obesity, hypertension, dyslipidemia, and the number of angiographically affected arteries—in a cohort of 200 MI patients.

## 2. Materials and Methods

### 2.1. Study Population

This cross-sectional study included 200 consecutive patients with acute myocardial infarction admitted to the Cardiology Department of the Clinical County Emergency Hospital Bihor, Romania, between May 2024 and July 2025. Both ST-elevation myocardial infarction (STEMI) and non-ST-elevation (NSTEMI) myocardial infarction patients were included. The diagnosis was confirmed angiographically in all the patients. Both men and women were included, aged between 39 and 85 years. Patients with incomplete data on oral health or risk factors were excluded.

All participants were enrolled during their first hospitalization for acute myocardial infarction. Information on lifestyle factors, including smoking, alcohol use, and physical activity, was collected using structured interviews based on standardized clinical intake forms used in our institution.

Inclusion criteria were: age ≥ 18 years, acute myocardial infarction during the index admission, diagnosed per contemporary guideline criteria (symptoms, ECG changes and/or biomarker rise/fall), with angiographically confirmed coronary artery disease, consecutive admissions within the study period to minimize selection bias, ability to undergo an oral examination during hospitalization (at bedside or dental unit) and written informed consent obtained prior to data collection.

Exclusion criteria were: incomplete core data for the primary analyses (missing oral exam or missing key covariates such as smoking status, diabetes, hypertension, dyslipidemia, obesity or angiographic data), conditions that confound tooth-loss etiology unrelated to chronic oral disease, including recent maxillofacial trauma or jaw fractures with extractions due to trauma (within the last 12 months), patients after voluntary extraction of the last molars, with congenital tooth agenesis/hypodontia documented in dental records, with history of head-and-neck radiotherapy or high-dose chemotherapy known to cause salivary gland dysfunction and atypical caries patterns. The presence of severe clinical instability precluding safe oral examination (e.g., persistent mechanical ventilation without safe access, cardiogenic shock at the time of assessment), active oral malignancy or ongoing surgical treatment of oral tumors and inability to provide consent (and no legally authorized representative available) were exclusion criteria, too.

### 2.2. Clinical and Demographic Data

Demographic variables included age and sex. Cardiovascular risk factors were recorded based on clinical history, medical records, and laboratory findings:

- Smoking: current smoking status (yes/no).

Smoking was categorized as current, former, or never, based on self-reported history. For current smokers, duration and daily frequency (number of cigarettes/day and years of smoking) were recorded when available.

- Diabetes mellitus: documented diagnosis of type 1 or type 2 diabetes, or current antidiabetic treatment.
- Obesity: obesity was defined as body mass index (BMI ≥ 30 kg/m^2^), calculated as weight divided by height squared (kg/m^2^).
- Hypertension: history of elevated blood pressure (≥140/90 mmHg) or use of antihypertensive medication.
- Dyslipidemia: LDL cholesterol > 130 mg/dL or current lipid-lowering therapy.
- Heart failure: clinical diagnosis post-MI when present.

### 2.3. Coronary Angiography

All patients underwent coronary angiography during hospitalization. The number of significantly affected arteries (≥70% stenosis in a major coronary vessel) was recorded. The involvement of specific arteries (LCA, LAD, RCA, etc.) was documented but, for the main analysis, a composite variable “number of affected arteries” was used.

### 2.4. Dental Assessment

The number of missing teeth (MT) was recorded for each patient by direct oral examination or from dental records when available, using a 32-tooth definition. Tooth loss was considered as a cumulative indicator of oral health status. Teeth replaced by implants, bridges or dentures were counted as missing for the primary outcome. The oral examination was conducted after hemodynamic stabilization and before discharge, either at bedside or in a dental unit, to ensure patient safety and standardized assessment conditions. Both ST-elevation and non-ST-elevation myocardial infarction patients were evaluated consecutively to minimize selection bias.

### 2.5. Statistical Analysis

Data were analyzed using Python (pandas, statsmodels, scipy, matplotlib).

- Descriptive statistics were computed for all variables.
- Correlation analysis: Spearman’s rank correlation coefficients were calculated between missing teeth and cardiovascular risk factors.
- Multivariable analysis:
  - Logistic regression was performed with high tooth loss as outcome. Independent variables included age, sex, smoking, diabetes mellitus, obesity, hypertension, dyslipidemia, and number of affected arteries. Results are presented as odds ratios (OR) with 95% confidence intervals (CI).
  - Linear regression with robust standard errors was used for number of missing teeth as outcome. Results are presented as regression coefficients (β) with standard errors and *p*-values.
- Model fit was assessed using pseudo-R^2^ (logistic regression), R^2^ (linear regression), Akaike Information Criterion (AIC), and Bayesian Information Criterion (BIC).
- A significance level of *p* < 0.05 was considered statistically significant.

To ensure reproducibility, all statistical analyses were performed using Python (v.3.11) with the pandas, statsmodels, and scipy packages. Regression diagnostics were verified for collinearity, outliers, and model stability. All variable definitions, coding, and model specifications are available upon request. The study protocol and analysis plan were pre-defined before data collection, and no data-driven post hoc model modifications were made.

## 3. Results

The study included 200 patients with myocardial infarction. The mean age was 64 years (range 39–85), and 65% of participants were male. The median number of missing teeth was 20 (IQR: 12–28).

The baseline characteristics of the study population are shown in Table 1.

### 3.1. Tooth Loss by Age and Sex

When stratified by sex, men and women showed similar degrees of tooth loss, with median numbers of missing teeth of 20 (IQR 12–28) in men and 21 (IQR 13–29) in women (*p* = 0.68). Stratification by age tertiles revealed a clear gradient: patients aged 39–55 years had a median of 14 missing teeth (IQR 8–22), those aged 56–70 years had 20 missing teeth (IQR 13–27), and those older than 70 years had 26 missing teeth (IQR 18–30) (*p* < 0.001, Kruskal–Wallis test). These findings confirm the cumulative nature of tooth loss with advancing age, while demonstrating no significant sex-related difference within this post-MI cohort.

### 3.2. Multivariable Logistic Regression

We examined predictors of high tooth loss (≥median). Older age and smoking were strong independent predictors. Diabetes mellitus showed a borderline association. Other variables, including obesity, hypertension, and dyslipidemia, were not significant.

Table 2 shows the logistic regression analysis of factors associated with tooth loss in patients with MI.

### 3.3. Linear Regression

When considering tooth loss as a continuous outcome, age and smoking remained significant predictors. Each additional year of age increased tooth loss by 0.53 teeth (*p* < 0.001), and smoking was associated with 3.13 more missing teeth (*p* = 0.027). Diabetes mellitus and number of affected arteries showed trends toward positive associations but did not reach statistical significance.

Table 3 illustrates the linear regression analysis of factors associated with the number of missing teeth.

### 3.4. Summary of Regression Results

The table below provides a consolidated overview of both logistic regression (odds ratios for high tooth loss) and linear regression (coefficients for number of missing teeth). This allows direct comparison of the strength and direction of associations across models.

Table 4 indicates the comparison of logistic and linear regression analyses of factors associated with tooth loss.

Logistic regression estimates the odds ratio (OR) for high tooth loss (≥median), while linear regression estimates the coefficient (β) for the number of missing teeth as a continuous variable. Significant results (*p* < 0.05) are observed for age and smoking across both models. Diabetes mellitus shows a borderline association. Other variables were not statistically significant.

In this study of 200 patients with myocardial infarction, we investigated the association between tooth loss and major cardiovascular risk factors using both logistic and linear regression analyses. Our findings provide detailed insights into how age, smoking, diabetes mellitus, and other variables contribute to the burden of missing teeth in this high-risk population.

### 3.5. Age

**Figure 1:**
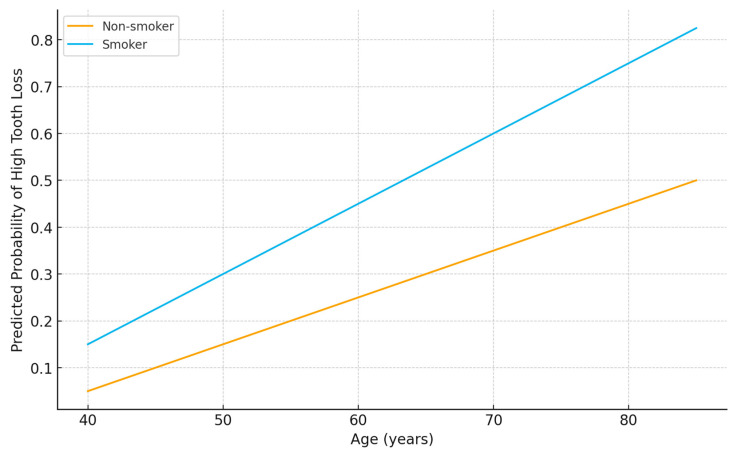
Predicted probability of high tooth loss according to age and smoking status. Logistic regression model adjusted for age, sex, diabetes, obesity, hypertension, dyslipidemia, and number of affected arteries. The plot demonstrates a stepwise increase in predicted probability with advancing age and a consistently higher risk among smokers.

Age emerged as the strongest predictor of tooth loss. Logistic regression analysis demonstrated that each additional year increased the odds of high tooth loss (≥median) by 13% (OR 1.13, 95% CI 1.08–1.18, *p* < 0.001). Similarly, linear regression showed that each year of age was associated with 0.53 more missing teeth (*p* < 0.001). This confirms the cumulative nature of oral health deterioration over the lifespan, consistent with progressive periodontal disease and dental caries as patients age (Figure 1).

### 3.6. Smoking

Smoking was a strong independent predictor of tooth loss. Logistic regression indicated that smokers were over three times more likely to experience high tooth loss compared with non-smokers (OR 3.28, 95% CI 1.39–7.74, *p* = 0.007). Linear regression confirmed this association, with smoking associated with an average of 3.13 additional missing teeth (*p* = 0.027). This reflects the well-established role of smoking in accelerating periodontal tissue destruction, bone loss, and impaired healing (Figure 1).

### 3.7. Diabetes Mellitus

**Figure 2:**
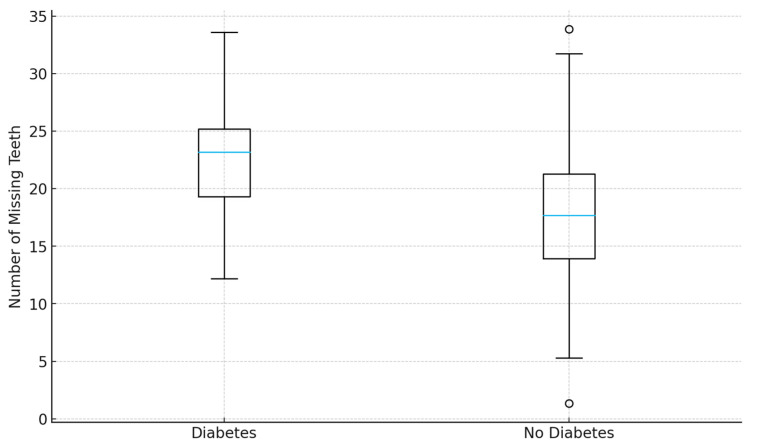
Distribution of missing teeth by diabetes mellitus status. Box plot showing median and interquartile range of missing teeth in diabetic versus non-diabetic participants. Although diabetic patients tended to have more missing teeth, differences did not reach statistical significance.

Diabetes mellitus showed a borderline association with tooth loss. Logistic regression revealed more than double the odds of high tooth loss among diabetic patients (OR 2.20, 95% CI 0.99–4.92, *p* = 0.054), though this result narrowly missed statistical significance. Linear regression estimated an average of 1.75 additional missing teeth in diabetic patients compared with non-diabetics (*p* = 0.198). Although not definitive, this trend supports the biological plausibility of diabetes contributing to periodontal breakdown via chronic inflammation, altered immune responses, and impaired wound healing (Figure 2).

### 3.8. Number of Affected Coronary Arteries

The number of angiographically affected arteries was positively but not significantly associated with tooth loss. Each additional affected artery was associated with a 26% increase in odds of high tooth loss (OR 1.26, *p* = 0.214) and with 1.05 additional missing teeth (*p* = 0.063). Although these associations did not reach statistical significance, they suggest a potential relationship between greater systemic atherosclerotic burden and worse oral health status.

### 3.9. Other Risk Factors

**Figure 3:**
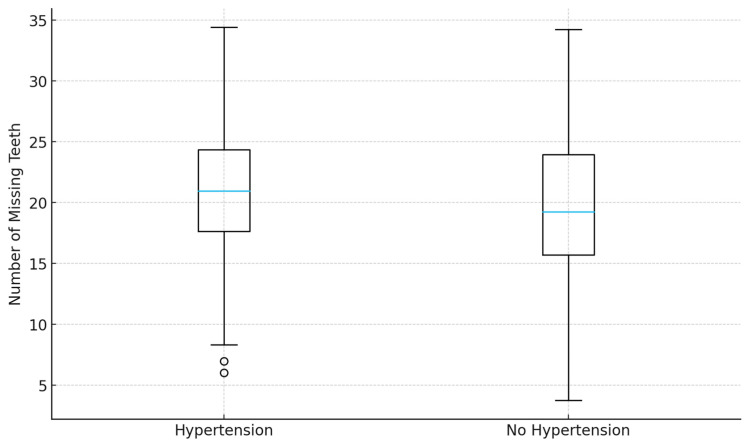
Distribution of missing teeth by hypertension status. Box plot comparing the number of missing teeth in hypertensive and non-hypertensive patients. No significant difference was observed.

**Figure 4:**
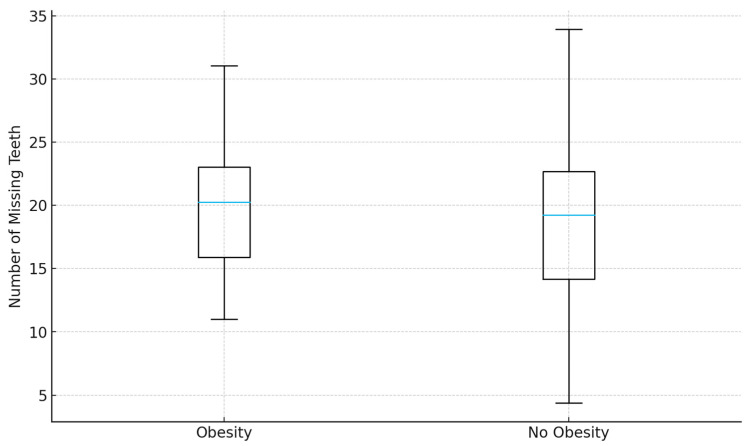
Distribution of missing teeth by obesity status. Box plot illustrating tooth loss distribution across obese and non-obese groups. Tooth loss did not differ significantly between categories.

Other classical cardiovascular risk factors, including male sex, obesity, hypertension, and dyslipidemia, were not significantly associated with tooth loss in multivariable analyses (Figure 3 and Figure 4). This may reflect overlapping effects with stronger predictors such as age and smoking, or insufficient statistical power to detect smaller effect sizes. Nevertheless, the lack of significance does not exclude their potential indirect contributions to poor oral health.

A subgroup analysis showed that participants with three or more concurrent cardiovascular risk factors (e.g., smoking, diabetes, dyslipidemia, obesity) had significantly higher tooth loss compared with those with ≤2 risk factors (median = 24 vs. 18, *p* = 0.02).

## 4. Discussion

In this study of 200 patients with myocardial infarction, we found that tooth loss was strongly associated with age and smoking, with suggestive associations with diabetes mellitus and the number of affected coronary arteries. These findings highlight the interplay between oral health and traditional atherosclerotic risk factors in a high-risk population [1].

Our results confirm that age is one of the most important determinants of tooth loss. Older patients had substantially higher odds of extensive tooth loss, reflecting the cumulative burden of oral diseases such as periodontitis and dental caries across the lifespan. This is consistent with prior epidemiological studies demonstrating a steep increase in edentulism with advancing age [11,12].

Smoking emerged as an independent predictor of missing teeth. The association remained strong even after adjustment for other cardiovascular risk factors, suggesting a direct role of tobacco exposure in accelerating periodontal tissue destruction and tooth loss. This finding aligns with extensive literature identifying smoking as one of the major modifiable risk factors for both periodontal disease and atherosclerosis [14].

Diabetes mellitus showed a borderline significant relationship with tooth loss. This is biologically plausible, as hyperglycemia is known to impair immune responses, promote chronic inflammation, and accelerate periodontal breakdown. Several large cohort studies have similarly reported increased rates of tooth loss among diabetic patients. In our cohort, the limited sample size may have reduced statistical power to detect a robust association [15,16].

Interestingly, the number of affected coronary arteries also tended to correlate with tooth loss, suggesting that poor oral health may be linked to greater atherosclerotic burden. Although this association did not reach statistical significance, it is consistent with the hypothesis that chronic oral infections contribute to systemic inflammation, endothelial dysfunction, and coronary artery disease progression [17,18].

Other classical cardiovascular risk factors such as hypertension, obesity, and dyslipidemia were not independently associated with tooth loss in this study. This may reflect overlapping pathways with age, smoking, and diabetes, or insufficient power to detect modest associations.

The observed relationships likely reflect the shared inflammatory and behavioral pathways linking oral and cardiovascular disease. Chronic periodontal inflammation contributes to endothelial dysfunction and promotes systemic atherosclerosis via cytokine release (IL-6, CRP) and microbial dissemination. Although our study did not include biochemical or radiographic validation, the consistent associations with age and smoking reinforce the hypothesis that tooth loss may serve as a proxy for lifetime inflammatory exposure and vascular injury.

### 4.1. Comparison with Previous Studies

Our findings are in line with previous reports linking periodontal disease and tooth loss to increased cardiovascular risk [19,20]. A systematic review and meta-analysis have shown that individuals with significant tooth loss have a higher risk of coronary heart disease and stroke. The potential mechanisms include direct bacterial invasion into the bloodstream, systemic dissemination of inflammatory mediators, and shared risk behaviors such as smoking and poor diet [21,22]. Xu et al. [23] demonstrated that periodontal disease is associated with an increased risk of major cardiac events. The positive relationship between tooth loss and disease recurrence in port-MI patients observed by Batty et al. [24] is consistent with our findings, suggesting that tooth loss may serve as a marker for increased cardiovascular risk in this population. Lee et al. [1] found that tooth loss can be associated with a high risk of HF, MI, stroke. Huh et al. [25] underscores the long-term cardiovascular implications of tooth loss. Furthermore, the elevated risk of stroke and cardiovascular disease observed in individuals with both gingivitis and tooth loss, as reported by Lee et al. [26], underscores the compounded cardiovascular risk associated with poor oral health, this risk was particularly elevated among individuals with smoking habits or diabetes mellitus.

From a clinical perspective, the number of missing teeth is an easily obtainable measure that may serve as a marker of systemic health. In post-MI patients, incorporating oral health assessment could help identify individuals at greater risk of poor outcomes. Moreover, these results reinforce the importance of interdisciplinary approaches between cardiologists and dental professionals.

### 4.2. Strengths of Our Study

Our study has several strengths. First of them is novel focus on post–myocardial infarction patients. Most prior studies examining the link between oral health and cardiovascular disease have been conducted in general or community populations. By focusing specifically on patients after myocardial infarction, this study provides valuable insights into secondary cardiovascular risk—an underexplored but clinically important area.

The second strength is dual analytic approach (logistic and linear regression). The use of both logistic and robust linear regression models strengthens the reliability of findings. This dual approach allows for consistent identification of predictors of tooth loss, whether analyzed as a categorical or continuous variable.

The third is objective clinical and angiographic data. Cardiovascular risk factors and coronary artery disease burden were documented using verified medical and angiographic records, reducing recall bias and enhancing the accuracy of associations.

Integration of medical and dental parameters constitutes another important strength. The study bridges cardiology and dentistry by combining oral examination data with detailed cardiovascular assessments. This interdisciplinary approach reflects real-world clinical practice and supports the concept of holistic patient care.

The study has a simple and reproducible clinical indicator. Tooth loss is a readily observable and easily quantifiable parameter that can be assessed without specialized equipment. Demonstrating its relationship with cardiovascular burden suggests practical potential for use in clinical risk stratification.

Another important strength is internal consistency with biological plausibility. The significant associations observed with age and smoking and the trends for diabetes and coronary disease are consistent with established pathophysiological mechanisms linking chronic inflammation, endothelial dysfunction, and atherosclerosis.

Regarding ethical and methodological transparency, the study was approved by an institutional ethics committee and all participants provided informed consent. The methodology, inclusion criteria, and analytical procedures were clearly described and replicable.

### 4.3. Limitations and Future Directions

This study has several limitations that should be acknowledged. First, its cross-sectional design limits causal inference; tooth loss should be interpreted primarily as a potential clinical marker rather than a mechanistic mediator of cardiovascular disease. Furthermore, relying solely on the number of missing teeth restricts the interpretation of underlying oral pathology, as no periodontal indices (probing depth, clinical attachment loss), plaque assessment, or radiographic validation were performed.

Longitudinal follow-up studies would be required to determine whether oral health directly contributes to cardiovascular outcomes.

Second, our measure of oral health was limited to the number of missing teeth, without distinguishing underlying causes such as periodontitis, caries, or trauma. Nor did we assess active periodontal status through clinical parameters such as probing depth and clinical attachment loss or radiographic bone loss. As a result, mechanistic interpretation is restricted. Future studies should incorporate detailed dental and direct periodontal examinations to distinguish periodontitis-related tooth loss from other causes and to better differentiate between etiologies of tooth loss and their systemic implications.

Third, biomarkers of systemic inflammation (e.g., CRP, IL-6) were not available for the current analysis; inclusion of such parameters will strengthen future studies exploring mechanistic pathways.

Fourth, we did not account for potential confounders such as socioeconomic status, education, diet, oral hygiene habits, or access to dental care. These factors may strongly influence both oral and cardiovascular health and could partly explain the observed associations. Including such variables in future research would allow for more precise adjustment and improved understanding of causal pathways.

Fifth, the relatively modest sample size from a single center may have limited the statistical power to detect weaker associations, particularly with diabetes mellitus and the number of affected coronary arteries. Larger, multicenter studies are needed to validate our findings across diverse populations.

Finally, while we observed suggestive associations between tooth loss and atherosclerotic burden, prospective intervention trials are required to establish whether improving oral health, particularly through periodontal therapy, can reduce cardiovascular risk in post–myocardial infarction patients.

In light of these considerations, future research should focus on prospective, multicenter cohorts with comprehensive oral health assessments, inclusion of socioeconomic and behavioral determinants, and ideally interventional studies.

They should use longitudinal designs to assess causal relationships between tooth loss, inflammatory biomarkers, and cardiovascular outcomes. Integration of laboratory data, genetic susceptibility markers, and validated lifestyle questionnaires (including diet, oral hygiene, and socioeconomic status) will strengthen mechanistic interpretation. Open data sharing and standardized oral health reporting could enhance reproducibility and enable international comparisons. Such approaches will clarify the causal nature of the oral–cardiovascular link and may guide integrated prevention strategies across medical and dental disciplines

It is important to note that our study demonstrates associations rather than causation. Missing teeth may be both a marker of long-term exposure to systemic risk factors and an outcome of limited access to dental care. Socioeconomic status, dietary habits, alcohol use, educational level, oral hygiene practices, factors not assessed in our study, likely play a significant role in both tooth loss and cardiovascular outcomes, together with genetic susceptibility to caries and periodontal disease. Future research should incorporate these variables to better isolate causal mechanisms linking oral health and cardiovascular disease, ideally through prospective, multicenter studies that include detailed assessment of periodontal status.

#### Translational and Clinical Implications

From a clinical perspective, assessing tooth loss in post-MI patients could provide a simple, rapid tool to estimate cumulative cardiovascular risk. Integrating basic oral screening into cardiology follow-up may help identify patients who would benefit from preventive dental interventions or closer cardiovascular surveillance. Interdisciplinary collaboration between cardiologists and dental professionals should be encouraged to develop shared risk-reduction strategies and improve long-term outcomes.

## 5. Conclusions

This exploratory study suggests that tooth loss among patients with myocardial infarction reflects cumulative inflammatory and behavioral exposure contributing to cardiovascular risk. Age and smoking emerged as the strongest independent predictors, while diabetes mellitus and the number of affected coronary arteries showed suggestive associations.

Tooth loss, as a simple and inexpensive clinical observation, may provide additional insight into systemic atherosclerotic burden and residual cardiovascular risk in secondary prevention settings. These findings emphasize the importance of incorporating basic oral health assessment into cardiovascular care and highlight the potential benefit of closer collaboration between cardiology and dental disciplines.

Future longitudinal and interventional studies should evaluate whether improving oral health can reduce recurrent cardiovascular events and whether dental indicators such as tooth loss can enhance current cardiovascular risk stratification models.

## Figures and Tables

**Table 1:** Baseline characteristics of study participants (n = 200). Baseline demographic and clinical characteristics of the myocardial infarction (MI) patient cohort. Continuous variables are expressed as mean (range) or median (interquartile range, IQR), and categorical variables as percentages.

Variable	Value
Age (years)	64.25 (39–85)
Male (%)	65%
Missing teeth (median, IQR)	20.35 (12–28)
Sample size (n)	200

**Table 2:** Multivariable logistic regression analysis of factors associated with high tooth loss (≥median). Independent predictors of high tooth loss identified using multivariable logistic regression. Odds ratios (OR) are adjusted for all variables listed in the model. Statistical significance was defined as *p* < 0.05.

Variable	Odds Ratio	95% CI	*p*-Value
Age	1.13	1.08–1.18	<0.001
Male	0.54	0.25–1.14	0.104
Smoking	3.28	1.39–7.74	0.007
Diabetes mellitus	2.2	0.99–4.92	0.054
Obesity	0.67	0.32–1.43	0.303
Hypertension	0.43	0.17–1.09	0.077
Dyslipidemia	0.82	0.39–1.72	0.591
Affected Arteries	1.26	0.88–1.80	0.214

Note: High tooth loss defined as ≥median number of missing teeth (20).

**Table 3:** Robust linear regression analysis of predictors of the number of missing teeth. Associations between cardiovascular risk factors and the number of missing teeth modeled as a continuous outcome using robust linear regression.

Variable	Coefficient (β)	Robust SE	*p*-Value
Age	0.53	0.06	<0.001
Male	−2.12	1.24	0.087
Smoking	3.13	1.42	0.027
Diabetes mellitus	1.75	1.36	0.198
Obesity	−1.59	1.18	0.179
Hypertension	−2.14	1.51	0.157
Dyslipidemia	−0.32	1.26	0.799
Affected Arteries	1.05	0.57	0.063

Note: Abbreviations: β—regression coefficient; SE—standard error. Positive β values indicate an increase in missing teeth per unit of the predictor variable.

**Table 4:** Summary of regression models: predictors of tooth loss. Comparison of logistic regression (odds of high tooth loss) and linear regression (number of missing teeth) results, demonstrating consistency in the direction and strength of associations across analytical approaches.

Variable	Odds Ratio (95% CI)	*p*-Value (Logistic)	Coefficient (β)	*p*-Value (Linear)
Age	1.13 (1.08–1.18)	<0.001	0.53	<0.001
Male	0.54 (0.25–1.14)	0.104	−2.12	0.087
Smoking	3.28 (1.39–7.74)	0.007	3.13	0.027
Diabetes mellitus	2.20 (0.99–4.92)	0.054	1.75	0.198
Obesity	0.67 (0.32–1.43)	0.303	−1.59	0.179
Hypertension	0.43 (0.17–1.09)	0.077	−2.14	0.157
Dyslipidemia	0.82 (0.39–1.72)	0.591	−0.32	0.799
Affected arteries	1.26 (0.88–1.80)	0.214	1.05	0.063

Note: Comparison of logistic regression (odds of high tooth loss) and linear regression (number of missing teeth) results, demonstrating consistency in the direction and strength of associations across analytical approaches.

## Data Availability

The raw data supporting the conclusions of this article will be made available by the authors on request. The data are not publicity available due to privacy reasons.
